# Modeling the complex gene × environment interplay in the simulated rheumatoid arthritis GAW15 data using latent variable structural equation modeling

**DOI:** 10.1186/1753-6561-1-s1-s118

**Published:** 2007-12-18

**Authors:** Nora L Nock, Emma K Larkin, Nathan J Morris, Yali Li, Catherine M Stein

**Affiliations:** 1 Division of Genetic and Molecular Epidemiology, Case Western Reserve University, Cleveland, OH 44106-7281, USA

## Abstract

Rheumatoid arthritis is a complex disease that appears to involve multiple genetic and environmental factors. Using the Genetic Analysis Workshop 15 simulated rheumatoid arthritis data and the structural equation modeling framework, we tested hypothesized "causal" rheumatoid arthritis model(s) by employing a novel latent gene construct approach that models individual genes as latent variables defined by multiple dense and non-dense single-nucleotide polymorphisms (SNPs). Our approach produced valid latent gene constructs, particularly with dense SNPs, which when coupled with other factors involved in rheumatoid arthritis, were able to generate good fitting models by certain goodness of fit indices. We observed that Gene F, C, DR, sex and smoking were significant predictors of rheumatoid arthritis but Genes A and E were not, which was generally, but not entirely, consistent with how the data were simulated. Our approach holds promise in unravelling complex diseases and improves upon current "one SNP (haplotype)-at-a-time" regression approaches by decreasing the number of statistical tests while minimizing problems with multicolinearity and haplotype estimation algorithm error. Furthermore, when genes are modeled as latent constructs simultaneously with other key cofactors, the approach provides enhanced control of confounding that should lead to less biased effect estimates among genes as well as between gene(s) and the complex disease. However, further study is needed to quantify bias, evaluate fit index disparity, and resolve multiplicative latent gene interactions. Moreover, because some *a priori *biological information is needed to form an initial substantive model, our approach may be most appropriate for candidate gene SNP panel applications.

## Background

Advances in the technology used to interrogate variation in the human genome (e.g., 10 K, 500 K and other single-nucleotide polymorphism (SNP) chip panels) are rapidly generating vast amounts of genotyping data. However, to maximize the use of this information in unravelling the etiology of complex diseases such as rheumatoid arthritis (RA), statistical approaches are needed that simultaneously model multiple genes and multiple SNPs within a gene in a hierarchical manner that reflects their underlying role in a biological system(s). We used the Genetic Analysis Workshop 15 (GAW15) simulated RA data and the structural equation modeling (SEM) statistical framework, which solves systems of linear and non-linear equations, to test the overall fit of hypothesized "causal" RA model(s) formally by employing a novel latent gene approach that models individual genes as latent (not directly measurable) variables defined by multiple SNPs.

## Methods

### Statistical methods and notation in SEM

The statistical theory involved in SEM is extensive so we only present the general concepts and notation needed to follow this work. SEM comprises two general sub-models: 1) a *measurement model *that develops the relationships between the observed variables (indicators) and the latent (unobserved) variables; and, 2) a *structural model *that develops the relationships between the latent variables. The general form of the *measurement model *is as follows [1]:

**x **= **Λ**_**x **_**ξ **+ **δ**

**y **= **Λ**_**y **_**η **+ **ε**,

where

**x **= q × 1 is a vector of observed indicators in the exogenous latent variables (**ξ**);

**y **= p × 1 is a vector of observed indicators in the endogenous latent variables (**η**);

**ξ **= n × 1 is a vector of latent exogenous (independent) latent random variables;

**η **= m × 1 is a vector of latent endogenous (dependent) latent random variables;

**δ **= q × 1 is a vector of measurement errors for **x**;

**ε **= p × 1 is a vector measurement errors for **y**;

**Λ**_**x **_= q × n is a matrix of coefficients relating **x **to **ξ**; and,

**Λ**_**y **_= p × n is a matrix of coefficients relating **y **to **η**.

The general form of the *structural model *is as follows [1]:

**η **= **B η **+ **Γ ξ **+ **ζ**,

where

**B **= m × m is a matrix of path coefficients for latent endogenous variables (η);

**Γ **= m × n is a matrix of path coefficients for latent exogenous variables (ξ); and,

**ζ **= m × 1 is a vector that represents the errors or disturbances in η.

To simplify notation, we refer to λ_xi _and λ_yi _values collectively as λ_i _values or measurement model "loadings" and β_j _and γ_j _values as β_j _values or structural model "path coefficients".

In SEM, the null hypothesis assumes that if the conceptualized model were correct, the population covariance matrix of the observed variables, **Σ**, would be exactly reproduced by the covariance matrix determined by the model parameters, **Σ**(**θ**) (Ho: **Σ **= **Σ**(**θ**)). Thus, covariance-based SEM aims to test "causal" model theory by minimizing the difference between the sample covariance matrix (**S**) and the covariance matrix defined by the model parameters (**Σ**(**θ**)) using a fitting function. Maximum likelihood (ML) estimation fitting functions (F_ML_) such as the following, where *p *is the number of observed variables, are often used for global optimization but require the rigid assumptions of multivariate normality and independence of observations [1]:

**F**_ML _= log|**Σ**(**θ**)| + tr[**S Σ**(**θ**)^-1^] - log|**S**| - (p).

Thus, for models with non-normal variables, a weighted least squares (WLS) fitting function (F_WLS_) should be used to obtain unbiased estimates, standard errors and model tests [1,2]:

**F**_WLS _= [**ρ **- **σ**(**θ**)]' **W**^-1 ^[**ρ **- **σ**(**θ**)],

where:

**W**^-1 ^is the weight matrix for the residuals;

**ρ **is the vector of elements containing polychoric, tetrachoric, and polyserial correlations;

**σ**(**θ**) is corresponding vector from same-order implied matrix **Σ**(**θ**); and,

**θ **is the t × 1 vector of free parameters.

Variations to these fitting functions have been devised, including the robust weighted least-squares estimator (WLSMV), which allows for estimation of binary and categorical dependent variables [2,3], and a ML estimator robust to non-normality (MLF) [4].

### Data preparation and modeling procedures

First, we randomly selected a training (Replicate 64) and a validation (Replicate 46) data set from the 100 replicates simulated. In an attempt to satisfy the requirement for independence of observations, the data files were reconstructed by randomly selecting one case from each affected sib pair (*n*_1 _= 1500) and including all unrelated controls (n_2 _= 2000). The SNPs were coded assuming an additive genetic model (e.g., 1/1 = 0; 1/2 = 1; 2/2 = 2 where: 1 = wild type allele; 2 = variant allele). Gender and smoking were dichotomous (0 = males; 1 = females; non-smokers = 0; smokers = 1). Because IgM and anti-CCP (anti-cyclic citrinullated protein) values were only provided for cases, we arbitrarily set the values of these variables to zero for controls. Using the location of simulated risk loci provided in the answer key, we selected genotyped SNPs from the 10 K and chromosome 6 dense SNP chip panels upstream, downstream, and directly at (when available) the known location of each locus to build latent constructs for each gene. Because we did not explicitly know which SNPs were representative of each simulated locus, we also examined the linkage disequilibrium (LD) structure (Haploview v3.2) between the selected SNPs and performed factor analysis (FA) using SAS v8.2 (SAS Institute, Inc.) to help devise viable gene constructs. FA was performed independent of disease status (with only the SNP data) and was used to generate eigenvalues, inspect scree plots and factor patterns, and determine the proportion of average variance explained (AVE). AVE is an indicator of the communality or validity of the construct [5]. We also inspected LD and Pearson correlations to help confirm initial SNP selections, particularly when FA suggested the construct was less than valid (AVE < 0.50) [5] or more than one factor was emerging.

We then built the full model(s) by constructing measurement and structural model equations using the "causal" RA model information provided in the answer key. However, to obtain scale determinancy and model identification, one of the loadings (e.g., the SNP with the highest loading from the FA or, ideally, the SNP with the largest biological impact on gene function) must to be fixed to 1.0. When we were able to locate a SNP in the exact physical location of the simulated locus, we fixed that SNP's loading to 1.0. When the simulated locus fell between two SNPs, we arbitrarily selected one of them. We analyzed the models using the WLSMV estimator in Mplus v4.1 (StatModel, Inc.) and evaluated the overall fit.

The chi-square test evaluates whether the specified model is significantly different from the alternative model, which assumes the data are from a multivariate normal distribution with an unconstrained covariance matrix. However, it is affected by departures from normality and sample size (with substantially more power to falsely reject an acceptable model with large samples). Therefore, when categorical or non-normal dependent variables are modeled, a modified chi-square test [6] or other fit index robust to non-normality is needed. Although a plethora of alternative goodness-of-fit indices exists, we chose to evaluate only the following three. The root mean squared error of approximation (RMSEA) is an absolute fit index which represents dispersal of data to model discrepancy across degrees of freedom; and, a RMSEA value of less than or equal to 0.05 is believed to represent the boundary of acceptable fit [7]. The Comparative Fit Index (CFI) is an incremental fit index that it is independent of sample size and values exceeding 0.96 indicate acceptable model fit [8]. The weighted root mean square residual (WRMR) is a relatively new fit index that is believed to be better suited to categorical data. WRMR values less than 1.0 depict a good fitting model [7]. We also evaluated the coefficients and their standard errors.

## Results

Even though we did not have explicit knowledge of which SNPs corresponded to each of the simulated genes, we were generally able to construct viable latent variables by using SNPs upstream, downstream, or directly at (where available) the physical location of each locus. Using eigenvalue (>1.0) and proportion of variance explained (AVE > 0.50) criteria [5], the latent constructs we devised for Gene C (dSNP6_ 3432-3439; 3.82; 0.52), Gene D (dSNP6_3912-3920: 3.31; 0.61), Gene F (SNP11_388-391: 1.61; 0.53), Gene G (SNP9_183-187: 2.12; 0.56) and Gene H (SNP9_189-195: 2.69; 0.59) were valid but those for Gene A (SNP16_29-33: 1.51; 0.40) and Gene E (SNP18_268-271: 1.13; 0.38) were marginal at best. Because Locus B was located at the end of chromosome 8 and the closest SNPs genotyped (SNP8_440-442) were all upstream and not representative of the simulated locus, we decided not to evaluate Gene B.

**Figure 1:**
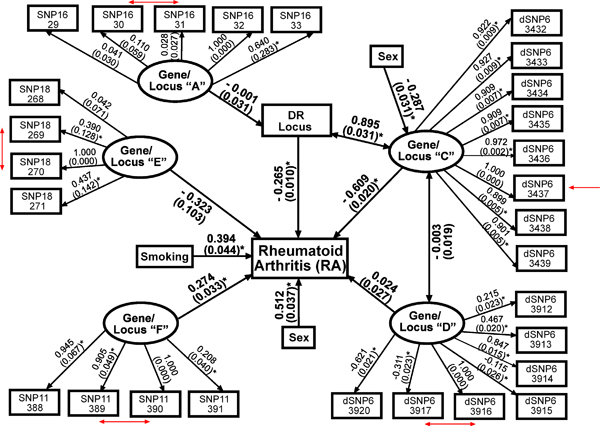
**Evaluation of the GAW15 simulated rheumatoidarthritis (RA) model**. Measurement model loadings depict relationships between observed variables (rectangles) and latent variables (ovals) and structural model path coefficients depict relationships between latent variables. Corresponding standard errors in parentheses are above single-headed arrows. Correlations are above double-headed arrows. Red arrows indicate the simulated locus location.*p-value ≤ 0.05.

We analyzed a "full" model with all genes (Gene A, C, D, E, and F), gender and smoking as covariates, and RA as a dichotomous outcome (Fig. 1) and obtained a good fitting model by CFI (0.96) but not RMSEA (0.12) or WRMR (6.53) fit index standards. To obtain convergence, we had to remove two SNPs (dSNP6_3918; dSNP6_3919) initially used in constructing latent variable Gene D. We could not determine the exact source of this problem but speculate it may have been due to some type of linear dependency between these SNPs because of the "weak" LD simulated between these loci. Nevertheless, removing the two SNPs did not alter the validity of Gene D (eigenvalue = 3.21; AVE = 0.59). The specific measurement model loadings and path coefficients for the "full" model are shown in Figure 1. The largest significant path coefficient between a gene construct and RA was observed with Gene C (β = -0.609 ± standard error of β = 0.020; *p *≤ 0.05) and the inverse nature of this association may reflect the increased risk simulated with the wild-type "C" allele. Gene C was also highly correlated with DR (ρ = 0.895 ± 0.031), which was expected given that Locus C was simulated to be in complete LD with DR (*D' *= 1.0). We also found a strong positive path coefficient between Gene F and RA (β = 0.274 ± 0.033; *p *≤ 0.05), which was expected because Locus F was simulated to confer risk from IgM on RA. However, the path coefficient between Gene D, which was simulated to be in "weak" LD with DR, and RA (β = 0.024 ± 0.027) was trivial in our model. We hypothesize the very low "D" risk allele frequency simulated contributed to this discrepancy. In addition, although the effects of DR were simulated to be controlled by Locus A, the path between Gene A and DR was negligible (β = 0.001 ± 0.022). This, however, may have been driven by our inability to devise a good construct for Locus A. When we added IgM (and/or anti-CCP), we did not obtain convergence, which was likely because of the skewed, edge effect distribution from assigning 0 values to controls.

We also evaluated RA severity as an ordinal outcome (1, 2, 3, 4, or 5) using all of the variables in the "full" model described above and Gene G and Gene H, which were simulated to induce RA severity. This was not a good fitting model by any index evaluated (CFI = 0.82; RMSEA = 0.19; WRMR = 10.73). Although the loadings and path coefficients of the Gene A, B, C, D, and F constructs were similar, the path coefficients between Gene G (β = -0.010 ± 0.031) and Gene H (β = -0.005 ± 0.130) and RA severity were surprisingly low, with high standard errors. Using just one of the RA severity genes (Gene G or H) did not materially alter the fit indices but removing both Gene G and H resulted in a model with a much better fit that was similar to that found with RA as a dichotomous outcome (CFI = 0.96; RMSEA = 0.12; WRMR = 6.53). These results suggest neither Gene G nor H (as described by our SNP selection) affected RA severity, which appears inconsistent with how the data were simulated.

We also examined subsets of the "full" model. In a "simpler" model including only latent gene constructs C and F, DR, sex and smoking (i.e., factors with significant paths on RA in the "full" model), the fit indices were poorer (CFI = 0.95; RMSEA = 0.25; WRMR = 12.02) and the path coefficients between Gene C and RA (β = 0.656 ± 0.015) and between Gene F (β = 0.324 ± 0.039) and RA were inflated by 7.71% and 18.25%, respectively, compared to the "full" model. The measurement model loadings for the Gene C and Gene F constructs, however, were not very sensitive to removal of the other gene constructs. The majority of other simpler models also resulted in poorer model fit and inflated path coefficients compared to the "full" model except for one that included only Gene C, Gene F, DR, sex, smoking and their paths on RA, which resulted in a good fitting model by CFI (0.96) and RMSEA (0.05) standards as well as a better fit by WRMR standards (2.45). We surmised the improvement occurred because the genes removed were not adding substantive information (Gene A, D, E) and because modeling Gene C and DR together made it difficult to define parameters representative of the data due to the complete LD (multi-allelic *D*' = 1.0) and complete linkage (recombination fraction = 0) simulated between locus C and DR. However, when Gene C was used in lieu of DR in a model also containing Gene F, sex, smoking, and their paths on DR, the model did not fit as well (CFI = 0.95; RMSEA = 0.27; WRMR = 13.94), suggesting DR was a better predictor of RA than Gene C.

We examined models with multiplicative latent variable gene interactions including a model with Gene A, DR, Gene A × DR and RA but the overall fit was very poor and the loadings and coefficients were very unstable. Other interaction models performed similarly.

Finally, we evaluated the "full" model depicted in Figure 1 using another randomly selected replicate (46) to serve as a *pseudo *model validation. The validation data set results were similar to the training data set; however, there was a little variation in the overall model fit indices and parameter estimates. The RMSEA (0.13) and WRMR (6.70) indices were similar but the CFI (0.92) was slightly lower in the validation data set. The most notable change in path coefficients was between Gene C and RA, which was slightly larger (β = -0.723 ± 0.019; *p *≤ 0.05).

## Discussion

Although we only had knowledge of the physical locations of the simulated loci and not biologically plausible SNP sets, we obtained valid latent gene constructs using dense SNPs, which appeared to perform well in full models (e.g., Gene C's loadings in Figure 1 are all large with small standard errors). The results using non-dense SNPs were mixed. For example, Gene A had poor validity by AVE standards [5] and its indicators had low loadings with large standard errors but the non-dense SNPs of Gene F all had large loadings with small standard errors and produced a valid construct.

When using SEM to evaluate the simulated RA "causal" model(s), we observed several models that had acceptable fit according to CFI and/or RMSEA but not WRMR criteria. CFI has been found to perform better than RMSEA, with a CFI value close to 0.96 providing acceptable rejection rates across models including those with binary outcomes such as RA when the sample size is ≥250 [9]. The "full" model depicted in Figure 1 and a "simpler" model with a subset of genes in the "full" model were evaluated using 3500 subjects and found to have a CFI of 0.96, which supports the conclusion that these models had good fit. Results from these models indicate that Gene F, C, DR, sex, and smoking were significant predictors of RA but Gene A and E were not, which is generally, but not totally, consistent with how the data were generated. However, because the power to detect misspecified loadings may be greater with the WRMR index [9], the measurement model loadings in the "full" and "simpler" models may be somewhat inaccurate. The inconsistency in model fit among these indices appears to be a general problem in SEM, which warrants further study using simulated multivariate SNP data over a range of LD.

## Conclusion

We conclude that our latent gene approach holds promise in unravelling complex diseases such as RA and improves upon current "one-SNP(haplotype)-at-a-time" regression approaches by decreasing the number of statistical tests while minimizing problems with multi-colinearity and haplotype estimation algorithm error. Furthermore, when genes are modeled as latent constructs simultaneously with other key cofactors, the approach provides for enhanced control of confounding that should lead to less biased effect estimates among genes as well as between gene(s) and the complex disease. However, further study is needed to quantify bias, evaluate fit index disparity and resolve multiplicative latent gene interactions. Because some *a priori *knowledge is needed, our approach may be best for candidate gene SNP panel data.

## Competing interests

The author(s) declare that they have no competing interests.
